# Nerve Regeneration with a Scaffold Incorporating an Absorbable Zinc-2% Iron Alloy Filament to Improve Axonal Guidance

**DOI:** 10.3390/pharmaceutics15112595

**Published:** 2023-11-07

**Authors:** Tomer Ron, Avi Leon, Alon Kafri, Ahmed Ashraf, John Na, Ashvin Babu, Runima Banerjee, Hunter Brookbank, Saimahesh Raju Muddaluri, Kevin J. Little, Eli Aghion, Sarah Pixley

**Affiliations:** 1 Department of Materials Engineering, Ben-Gurion University of the Negev, Beer-Sheva 8410501, Israel; 2 Nuclear Research Centre-Negev, Beer-Sheva 84190, Israel; 3 College of Medicine, University of Cincinnati, Cincinnati, OH 45267, USA; 4 School of Medicine, Case Western Reserve University, Cleveland, OH 44106, USA; 5 College of Engineering & Applied Sciences, University of Cincinnati, Cincinnati, OH 45221, USA; 6 College of Arts & Sciences, University of Cincinnati, Cincinnati, OH 45221, USA; 7 Department of Orthopedic Surgery, College of Medicine, University of Cincinnati, Cincinnati, OH 45267, USA; 8 Pediatric Hand & Upper Extremity Center, Cincinnati Children’s Hospital Medical Center, Cincinnati, OH 45229, USA; 9 Department of Pharmacology & Systems Physiology, College of Medicine, University of Cincinnati, Cincinnati, OH 45267, USA

**Keywords:** nerve regeneration, absorbable metal, metallic implant, zinc, magnesium, iron, alloy, peripheral nerve, sciatic nerve, rat

## Abstract

Peripheral nerve damage that results in lost segments requires surgery, but currently available hollow scaffolds have limitations that could be overcome by adding internal guidance support. A novel solution is to use filaments of absorbable metals to supply physical support and guidance for nerve regeneration that then safely disappear from the body. Previously, we showed that thin filaments of magnesium metal (Mg) would support nerve regeneration. Here, we tested another absorbable metal, zinc (Zn), using a proprietary zinc alloy with 2% iron (Zn-2%Fe) that was designed to overcome the limitations of both Mg and pure Zn metal. Non-critical-sized gaps in adult rat sciatic nerves were repaired with silicone conduits plus single filaments of Zn-2%Fe, Mg, or no metal, with autografts as controls. After seventeen weeks, all groups showed equal recovery of function and axonal density at the distal end of the conduit. The Zn alloy group showed some improvements in early rat health and recovery of function. The alloy had a greater local accumulation of degradation products and inflammatory cells than Mg; however, both metals had an equally thin capsule (no difference in tissue irritation) and no toxicity or inflammation in neighboring nerve tissues. Therefore, Zn-2%Fe, like Mg, is biocompatible and has great potential for use in nervous tissue regeneration and repair.

## 1. Introduction

Peripheral nerve injury (PNI) affects an estimated 5 million people worldwide due to both initial injuries and later disabilities leading to a loss of productivity [1]. PNI occurs in ~5% of emergency room visits for trauma, with other instances occurring due to surgical resection, as in tumor removal [2,3]. Based on estimates of ~560,000 surgeries per year to repair nerve injuries in limbs alone, the market for new and improved methods of repair was estimated to be USD 1.3–1.9 billion [4]. When PNI results in complete nerve transection, surgical repair is needed to reconnect nerve stumps and, if direct nerve reconnection is not possible, then a scaffold is used to replace the lost segments [5,6]. Autografts, the current gold standard of repair, can replace the longest possible injury gaps to date, but they also result in a second site of morbidity, present issues of size and fiber-type mismatch, and still do not support full, functional recovery [4,7,8]. Processed human allografts are promising; however, they still cannot match autograft repairs and they contain human materials that present an immunological risk [4,7,8]. Therefore, research efforts continue to improve hollow nerve conduits made of non-human materials. Some conduits are in clinical use, however they only supply functional recovery comparable to that achieved through autografts for nerve gaps of less than ~20 mm in humans [4,7,9,10]. One promising factor that can increase the gap length repaired by hollow conduits is including an internal scaffold that supplies physical contact guidance for cells to cross gaps [7,9,10,11,12]. None of the many configurations tested to date has yet reached clinical implementation, so research is needed to identify improved internal support materials.

Absorbable metals are exciting materials for tissue engineering because they prevent long-term tissue irritation and avoid the need for surgical removal. The absorbable metals magnesium (Mg), zinc (Zn), and iron (Fe) have been shown to be safe when used for hard tissue repair or as stents to keep circular organs open [11]. Because these metals release beneficial metal ions during degradation, they are also efficacious for soft tissue repairs. Our lab has previously shown that single Mg filaments (250–300 µm diameter) placed inside nerve conduits provided physical support, allowed cellular attachment, improved aspects of short-gap nerve growth, left no scar after degradation, and reduced tissue inflammation [12,13,14]. Others have shown that increasing systemic or local tissue levels of Mg ions improved nerve regeneration after a nerve crush [15,16,17].

In this work, we proposed that absorbable filaments made with a base of Zn would be biocompatible and have advantages over Mg in nerve repair. The potential advantages of Zn include a slower in vivo degradation rate (better for support across longer nerve gaps, an issue that we encountered with Mg), less reactivity to water (Mg degradation produces hydrogen bubbles that can disrupt tissue attachment), and that there are greater anti-inflammatory and antisepsis effects of Zn versus Mg ions [18,19,20,21,22,23,24]. Our studies on Mg filaments for nerve regeneration showed that the Mg filaments developed gaps by six weeks in vivo, which we speculated would be too soon to provide sufficient physical support for longer injury gaps [12,13,14]. We saw that Mg filaments improved regeneration across short gaps (6 mm), but not 15 mm gaps, which is a critical size for gaps in rats and requires the metal to remain intact longer [13]. However, the challenges with pure Zn include that the degradation rate in vivo may be too slow for use in nerve regeneration [25,26,27]. Pure Zn filaments (of a size similar to our already tested Mg filaments) remained intact after implantation in animal arterial tissues for at least 3–4 months and retained 60% of their structure after a year [27,28]. The time that a scaffold must remain intact for nerve repair is weeks rather than months, as non-neurons form a tissue strand across a 10 mm gap inside a silicone conduit by three weeks and axons cross by four weeks [29]. The retention of the metal inside the naturally restricted space of a peripheral nerve for a year would limit the number of axons regenerating and cause tissue friction and damage. We proposed that the degradation rate needed to be between that of pure Mg and that of pure Zn. To speed up Zn degradation, our team developed new proprietary alloys of Zn containing Fe that showed more rapid degradation rates than pure Zn does in vitro and in vivo, with no local, systemic, or organ toxicity after pellets of the metals were implanted under the skin in rodents [30,31,32]. As regenerating axons are highly sensitive to toxicity, and some toxicity has been seen when using Fe [27,28], our goals in this study were to determine if filaments of the Zn-2%Fe alloy would not only support nerve regeneration but were also biocompatible and nontoxic. To our knowledge, neither Zn nor any Zn alloy has ever been used for this type of application in nerve repair. We report here that both pure Mg and Zn-2% Fe alloy filaments placed inside silicone nerve conduits to repair a 6 mm gap were very compatible with nerve regeneration and supported abundant axonal growth.

## 2. Materials and Methods

### 2.1. Zn-2%Fe Alloy

A proprietary Zn alloy was produced by placing pure zinc bars (99.99%) and pure iron (99%) powder (325-mesh) in the desired amounts in a graphite crucible and heating them to 750 °C for 3 h. To obtain a homogenized alloy, the melt was stirred every 30 min. The chemical compositions of the obtained ingots were determined using an Inductively Coupled Plasma Optical Emission Spectrometer (ICP-SPECTRO, ARCOS FHS-12, Kelve, Germany). The Zn-2%Fe chemical composition was as follows (in wt%): Fe: 1.919; Ca: 0.003; Cu: 0.002; Al: 0.001; Mg: 0; Pb: 0.003; balance Zn. A gravity casting technology using rapid solidification conditions was used to make castings that were rods with 9 or 15 mm diameters.

### 2.2. Preparation of Thin Zn Filaments

The preparation of the Zn-based microfilaments included the following production steps: (i) machining the casted 15 mm diameter rods to obtain 13 mm diameter rods that were required for the extrusion process, (ii) hot extrusion processing at 400 °C to obtain 3 mm diameter wires, and (iii) electropolishing to obtain microfilaments with the desired diameter of about 300 µm. The electropolishing procedure was based on the process developed by Guillory [33], with some modifications. The electropolish solution contained 62 gr anhydrous zinc chloride, 27 gr aluminum trichloride hexahydrate, 320 mL ethyl alcohol, and 30 mL deionized water. We applied a voltage of 10 V and a current of up to 1 A. The filaments with the final 300 µm diameter were cut to ~1 cm lengths for placement in the scaffolds. Pure (99.9%) Mg filaments of 300 µm diameters were purchased from Goodfellow, USA, and used as received.

### 2.3. Zn Metal Characterization

The corrosion performance was evaluated through the immersion testing of pellets of a 9 mm diameter and 8 mm height for up to 10 days according to the ASTM G31–12a, in a standard phosphate-buffered saline (PBS) solution at 37 °C. The surface of the Zn-based microfilaments was analyzed using scanning electron microscopy (SEM) (SEM JEOL 5600, JEOL Ltd., Tokyo, Japan) and the corrosion rate was normalized to the dimensions according to standards.

### 2.4. Preparation of Scaffolds for Surgery

**Figure 1:**
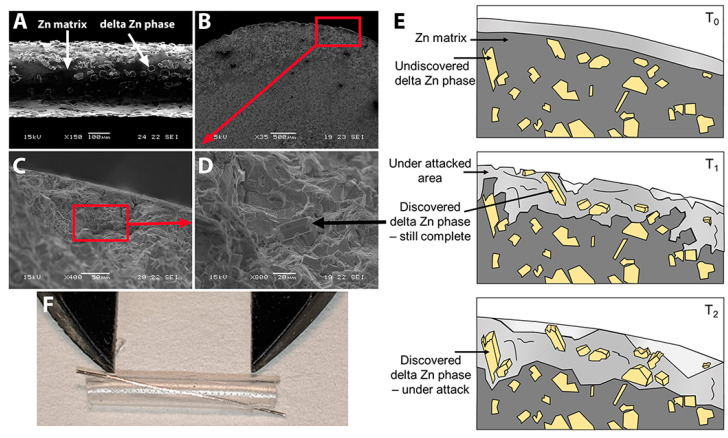
**The Zn alloy.** (**A**) Zn-2%Fe alloy filament (300 µm diameter) after electropolishing (bar = 100 µm). Arrows show the Zn matrix and delta Zn phase (Zn_11_Fe). (**B**–**D**) Effects of corrosion attack after 10-day immersion in PBS. Bar in B = 500 µm, C = 50 µm, D = 20 µm. Black arrow in D points to one delta Zn phase precipitant. Red boxes show representative areas magnified in the next image. (**E**) Schematic illustration of the degradation mechanism of Zn-Fe alloys over time. (**F**) Zn-2%Fe alloy filament placed in silicone nerve conduit; calipers set at 6 mm.

The conduits were silicone (Thermo Fisher Sci, St. Louis, MO, USA) with an ID of 1.46 mm and an OD of 1.96 mm, cut to a length of 10 mm. A single Mg or Zn-2%Fe filament was inserted through the wall of the silicone conduit at one end and out the other wall, at an angle, leaving 6 mm of metal exposed inside the conduit (Figure 1F). Excess external metal was cut off. The scaffolds were sterilized via immersion in 70% ethanol, dried under sterile conditions, and placed in sterile tubes to take to surgery.

### 2.5. Animal Use and Surgery

A total of 29 young adult male Lewis rats (weights at surgery: 227 +/− 8.7 g) were used for this work. All protocols for surgery, animal care, and euthanasia were approved by the University of Cincinnati (UC)’s Institutional Animal Use and Care Committee (IACUC) and all personnel were appropriately trained. Animals were housed in AAALC-approved facilities with strict lighting and humidity controls, and full-time veterinarian and husbandry support. For surgery, the techniques used were those previously described [13]. In brief, animals were anesthetized (isoflurane gas with vacuum retrieval); they then received subcutaneous injections of an analgesic cocktail of Rimadyl^TM^ (carprofen, 5 mg/kg) and buprenorphine SR (slow release, 1 mg/kg). Additional Rimadyl injections were administered every 12 h for 48 h. The surgical site was shaved, eye lubricant was applied, animals were placed on warming pads, and the site was prepared for sterile surgery. The sciatic nerve was exposed, 6 mm of nerve was removed, nerve stumps were allowed to retract, and repairs were made. For autograft animals (positive control), the removed nerve section was reversed and re-attached with two epineurial 9-0 Ethilon sutures (Ethicon, Inc., Johnson & Johnson, Raritan, NJ, USA). For all conduit repairs, ~2 mm of the proximal and distal nerve stumps was pulled into the conduits, contacting metal if present, creating gaps of 6 mm, and secured to the conduit with two 9-0 epineurial sutures. Conduits were filled using a syringe with sterile Dulbecco’s Modified Eagle’s Medium (DMEM, high glucose with pyruvate and phenol red, Corning brand, Thermo Fisher Scientific, Waltham, MA, USA). The Mg-group animals were infused with either DMEM or sterile, pharmaceutical-grade 2 M magnesium sulfate (Fresenius Kabi, Lake Zurich, IL, USA). As the two Mg groups did not differ, data for the Mg/DMEM (n = 6) and Mg/Mg sulfate (n = 5) groups were combined into one Mg group (n = 11). Muscle and skin were closed with 4-0 Vicryl sutures (Ethicon) and metal staples, respectively. Lidocaine gel was applied to the wound and the animals were warmed until mobile and returned to clean cages. Four animals died during the first week after surgery. The resulting 25 animals were in the following groups: (1) Aut: autografts (n = 5); (2) Em: conduits (empty, negative control) (n = 5); (3) Mg: conduits with a single Mg filament (n = 11); and (4) Zn: conduits with a Zn filament (n = 7).

### 2.6. Live Animal Functional Monitoring

Animal function was assessed at ~weekly intervals over 17 weeks, using the noninvasive tests described previously [12,13,14] or shown in Figure S1; scoring criteria are shown in Table S1. Measures collected were weight, the circumference of each hind leg at the level of the belly of the calf muscle, and a pinch test of the skin on the ankle and lateral foot and two areas on the lateral (#5) toe. The pinch test used blunt forceps and reactions were compared to those of pinch tests on the uninjured foot. Reflex toe, foot, and leg movements were monitored after lifting the hind limbs off the surface. The measures of toe spread (TS), toe contractures (Ctrs), and foot extension (Ext) were analyzed by three blinded observers using a scoring system before week 11 and via photographs after week 11. The raw data are shown in Figure S2, which also shows a cut-off level separating “positive” from “negative” scores. A Yes/No score was assigned, and the percentage of animals per group per week post-surgery (WPS) was calculated.

### 2.7. Sacrifice and Tissue Preparation

After animal euthanasia via approved protocols, the conduits plus 3–4 mm of proximal and distal nerve stumps were removed, as were similar lengths of control nerves and autografts. The distal ~2 mm of the conduits plus distal stumps, and comparable sections of autograft nerves, were removed and fixed in glutaraldehyde (see below). The proximal portions, including ~8 mm of the conduits and proximal nerve stumps or equivalent tissues, were fixed in 4% paraformaldehyde (pH 7.4 in PBS) for 24 h (4 °C), and rinsed in PBS. After removing nerves, the calf muscles (triceps surae = medial and lateral gastrocnemius heads plus soleus) were dissected out of both hind legs and weighed.

### 2.8. Micro Computed Tomography (Micro-CT)

After paraformaldehyde fixation, soft tissue contrast was provided by emersion in Lugol’s iodine solution and then micro-CT imaging of axial stacks was performed as described previously [14,34,35]. Stacks were exported as DICOM files, as described [14]. Images were viewed and analyzed with the public domain image JAVA processing program, ImageJ.

### 2.9. Histology: Tissue Preparation

*Glutaraldehyde-fixed distal tissues:* The distal sections of nerves were fixed in 3% glutaraldehyde in 0.15 M sodium cacodylate buffer, processed through a series of alcohols, infiltrated, and embedded in LX-112 resin. After polymerization at 60 °C for three days, thin sections (1–2 µm) were cut using a Leica Em UC7 ultramicrotome and stained with 1% toluidine blue.

*Paraformaldehyde-fixed proximal tissues*: After micro-CT imaging, proximal tissues were cleared of iodine with 2.5% sodium thiosulfate in PBS [34]. The tissues were cut into proximal, middle, and distal pieces and embedded in one paraffin block, and cross sections were cut at 7 µm on a standard microtome and mounted on slides. After dewaxing and rehydrating, sections were stained with hematoxylin and eosin (H&E) or double-immunostained for axons (anti-200 MW neurofilament protein, Sigma Aldrich, St. Louis, MO, USA) and functionally active perineurial fibroblasts (the perineurium, anti-glucose transporter protein-1, Sigma) and nuclei, as described previously [12].

### 2.10. Analysis of Axon Density

Cross sections of toluidine nerves at the distal ends of the conduits and autografts were photographed and images were stitched together using a Leica DMi8 bright-field microscope with a color camera (Leica, Germany, 4×, 10×, or 40× objectives). Images where the stain did not take (a small number) were photographed using a Zeiss microscope with a Zeiss Axiocam color camera (Zeiss, Germany, 100× objective). The density of axons in the fascicles was calculated by three blinded observers using random sampling and stereological principles [36]. In brief, ImageJ was used to place a grid pattern (150 µm square) randomly over nerve sections, and every other grid was chosen, with at least 25 grids sampled per section per animal. Using the LAS X software (Leica), regions of interest (ROI = 75 µm square) were chosen in the center of each selected grid. Axons were counted if the top point of the outer myelin sheath was within the grid and, if the top spanned two images then only nerves crossing two of the four sides (sides predetermined) were counted [36]. The average density per rat section was used for statistics. One autograft animal was cut longitudinally so average density was not calculated. Photoshop was used to prepare figures. Adjustments to brightness and contrast were used for clarification during counting and illustration in figures but they did not affect axon counts.

### 2.11. Analysis of Nerve Histology

All H&E-stained tissue sections with cavities where metal had been present at euthanasia were imaged as above and capsule thickness and cavity diameters were measured using ImageJ and/or LAS X. Cavities left by the loss of metal during tissue processing were more abundant in the Zn group (found in five of the six Zn animals) than in the Mg group (n = 3), as expected from the micro-CT imaging, which showed the loss of most of the Mg metal. For capsule thickness, the inner edge of the circumferentially oriented connective tissues (fibroblasts and collagen or extracellular matrix (ECM) materials) was identified. Radial lines were drawn to the nearest place with tightly packed nerve fascicles (6 measurements per image, 2–5 images per rat). Scattered myelinated axons found in the capsule were not considered nerve tissue for these measurements. The diameters of cavities were measured between the inner edges of the capsule at the thinnest diameter of the cavity (to account for oblique angles of filament insertions). Capsule or diameter measurements were not taken where the inner capsule contacted the outer tissue capsule of the tissue strand or protruded into the conduit, or where the section was cut obliquely (i.e., not a cross section).

### 2.12. Statistics

SigmaPlot (Inpixon, Palo Alto, CA, USA) was used to calculate normality and equal variance. Normally distributed data were analyzed using an analysis of variance (ANOVA) test and the Holm–Sidak or Student–Newman–Keuls multiple-comparisons post hoc tests, and they were graphed using mean +/− standard deviations (SD). Non-normal data were analyzed using a Kruskal–Wallis ANOVA followed by Dunn’s post hoc test, comparing all combinations, and were graphed with box plots using medians +/− 25th and 75th percentiles. A *p* value of <0.05 was considered significant. For functional measures analyzed using nominal scales, data are described qualitatively.

## 3. Results

### 3.1. The Zn-2%Fe Alloy Filaments and Preparation for Surgery

The corrosion rates of metal pellets immersed in PBS for 10 days were higher for Zn-2%Fe (0.47 +/− 0.04 mm/year) than for pure Zn (0.21 +/− 0.01 mm/year, n = 3 pellets each). In preparation for implantation, filaments with a 300 µm diameter were made of the Zn-2%Fe alloy, using electropolishing as the last step. Figure 1A shows an SEM image of the surface. The electropolishing exposed the delta phase (Zn_11_Fe) in the Zn matrix and created a rough surface. Immersion in a PBS solution for 10 days similarly exposed the delta-phase particles, as shown in Figure 1B–D. The arrow in Figure 1D points to one exposed particle. The diagram in Figure 1E outlines the degradation process. Prior to exposure to a corrosive environment at T_0_, the Zn matrix was intact and unaffected. However, as exposure time increased to T_1_, the Zn matrix began to degrade preferentially, and the inner delta phase started to appear. At this point, the second phase remained undamaged but functioned as a micro-galvanic interface with the Zn matrix, increasing the corrosion rate. Once the exposure time reached T_2_, the second phase started to degrade as well. In preparation for surgical repair and implantation, a single Zn-2%Fe filament was secured inside a silicone nerve conduit as shown in Figure 1F, with 6 mm of metal exposed inside the conduit.

### 3.2. Early Rat Health

**Figure 2:**
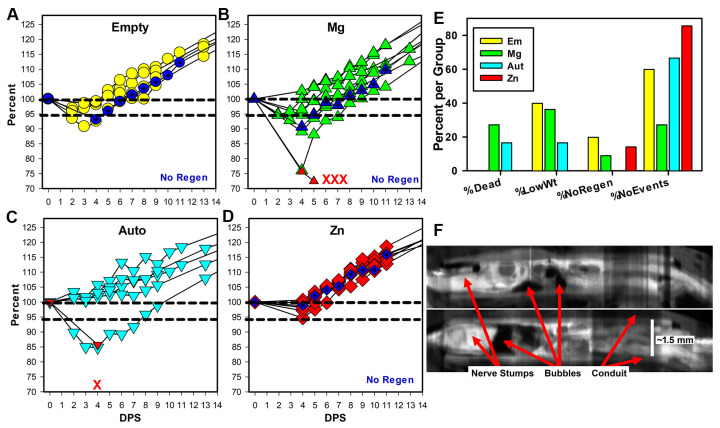
**Early animal health.** (**A**–**D**) Weights are shown as a percentage of initial surgery weight per days post-surgery (DPS) for the first two weeks. Symbols in dark blue and the label No Regen indicate animals that did not have tissue regeneration at 17 weeks. Animal deaths per group are represented by the red X marks, and for two animals, weights were taken before death (red upward triangles in (**B**,**C**)). (**E**) The graph shows the percentage of animals/group that died, had weights below 95% of surgery weight (%LowWt), had no tissue inside conduits (%NoRegen), and had none of these events (%NoEvents). (**F**) Composite longitudinal views are shown of conduits and tissues from two Mg animals that died, imaged using micro-CT with iodine contrast. Tissues are white, conduits gray, and air or water black. Magnification is based on the known internal diameter of the conduit.

The use of Zn resulted in fewer adverse rat health events over the first two weeks after surgery, as shown in Figure 2A–E and Table S2. Adverse events included rat deaths during the first week after surgery (4 rats/29; 3 in the Mg group and 1 in the Aut group), loss of weight during the first week after surgery, and a lack of regeneration through the conduits. Deaths, which we had not observed in previous experiments, appeared due to sepsis/multiorgan failure. Weight loss was a loss of weight below 95% of surgery weight. The lack of tissue regeneration was determined through micro-CT imaging after euthanasia at 17 weeks but is included here as it presumably happened fairly early after surgery. A lack of regeneration (No Regen) occurred for three animals (one each in the Em, Mg, and Zn groups). For the Zn animal only, visual inspection at euthanasia showed that the nerve had not entered the conduit (or retracted) and nerve tissue was attached, instead, to the outer surface of the proximal conduit, and did not extend to the distal conduit. The percentages of animals per group with each event and then without any events are shown in Figure 2E, and the numbers are shown in Table S2. The percentage of animals per group without any events was Zn (85%) > Aut (66.7%) > Em (60%) > Mg (27.3%). The conduits were retrieved for two of the Mg animals that died early, and micro-CT imaging with iodine contrast showed the presence of what were most likely hydrogen gas bubbles with low density (dark in CT) inside the conduits (Figure 2F). When Mg degrades in a salt solution, it creates bubbles of hydrogen gas, and these appear to have been trapped inside the conduit. After the second week post-surgery (WPS), there were no differences in weights between groups at any other time point (Figure S3), suggesting that health issues had been resolved. No correlation was detected between the lowest weights after surgery and any of the functional measures.

### 3.3. Functional Changes over Time

**Figure 3:**
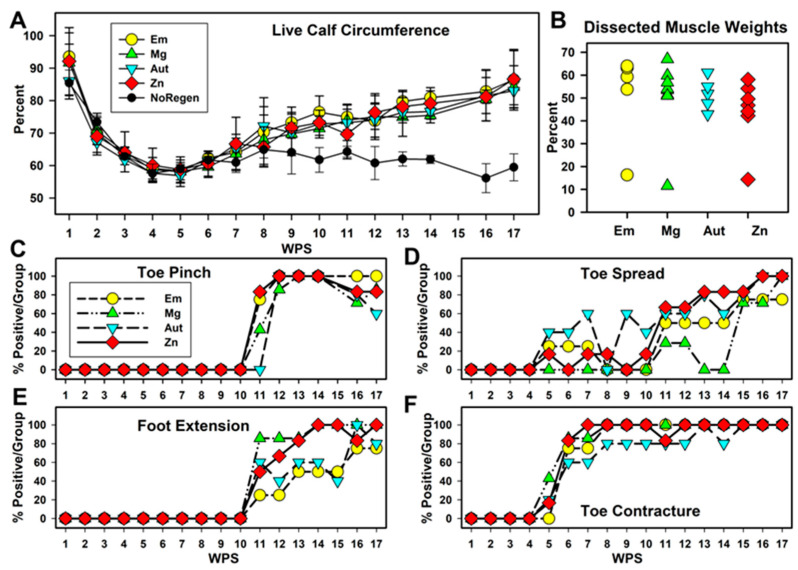
**Animal function.** (**A**) Calf circumference in live animals (injured as a % of control leg). Animals without tissue regeneration are graphed separately (NoRegen, black circles). (**B**) Weight of calf muscles after euthanasia (injured as % of control muscle). (**C**–**F**) Percentage of rats positive for each measure per group per timepoint for (**C**) toe pinch, (**D**) reflex lateral toe spread, (**E**) fixed foot extension of lower leg vs. thigh, and (**F**) fixed contraction of toes (toe contractures).

When the sciatic nerve is severed, the calf muscle atrophies, and atrophy can be reversed upon nerve regeneration. To monitor calf muscle atrophy, the circumferences of both calves on the hind legs were measured. This is expressed in Figure 3A with the injured calf as a percentage of the uninjured calf. Calf circumference decreased rapidly after surgery with no differences between groups, to a minimum average of ~60% by WPS 5. Calf muscles recovered to levels around 85% by WPS 17 in all animals except the three animals without tissue regeneration (grouped separately as NoRegen, black circles in Figure 3A). Analysis (two-way repeated-measures ANOVA (*p* < 0.001 for interactions, group, and WPS)) showed that there were no differences between the Em, Mg, Aut, and Zn groups at any timepoint, but they differed from the NoRegen group at WPS 10 and 12 through WPS 17. The groups with regeneration increased significantly over the low WPS 5 values at WPS 9 through 17; however, no differences were seen for the NoRegen group. After euthanasia, the calf muscle weights, which are more definitive of muscle atrophy recovery, returned to between 40 and 70% of the control leg, while the NoRegen animals had values between 10 and 15%. Weights as percents (omitting the NoRegen animals) showed a significant difference between groups (ANOVA, *p* = 0.045, n = 4, 7, 6, and 5 for Em, Mg, Aut, and Zn groups), but no differences were found in the Holm–Sidak post hoc test, although Em was greater than Zn with a *p* value of 0.064.

Other functionally relevant phenomena were measured using nominal scales (Figure S1 and Table S1). The raw scores are graphed in Figure S3; cut-off levels were assigned to provide Yes/No values, and the percentage of animals positive for the function per group per WPS is charted in Figure 3. Sensation return was monitored with a pinch test of the #5 lateral toe (Figure 3C). Toe sensation appeared at WPS 11 for the three conduit groups, but not until WPS 12 for the Aut group. Between WPS 12 and 16, almost all rats showed toe responses, and declines were seen starting at WPS 16. Pinch tests of the ankle and lateral foot skin (Figure S2E) also appeared between WPS 11 and 12 for all but the NoRegen animals. This foot sensation was speculated to be an expansion of the territory of the uninjured saphenous nerve that usually supplies the medial foot. Motor function was monitored through the reflex spreading of the lateral toe after lifting the hind end (toe spread, TS), and percentages are shown in Figure 3D. Some animals, with higher percentages in the Aut group for some weeks, showed positive toe spread between WPS 5 and 10, but values were highly variable. Beginning at WPS 11, the Zn, Aut, and Em groups showed similar steady increases, while the Mg group remained variable until WPS 15, and all groups reached their maximum over the last three weeks. No animal achieved a normal toe spread.

Two fixed and involuntary phenomena appeared over time; foot extension past 90 degrees relative to the long axis of the thigh (Figure 3E, Figures S1 and S2; scores in Table S1) and toe contractures (Figure 3F, Figures S1 and S2; scores in Table S1). These changes indicated partial motor re-innervation (i.e., flexors re-innervated but not extensors). Foot extensions first appeared at WPS 11 (as did pinch values). Between WPS 11 and 15, extensions were more common in the Mg and Zn groups than in the Em and Aut groups. All groups approached 100% between WPS 16 and 17. Toe contractures (Figure 3F) appeared at WPS 5–6 in all groups (as did toe spread values). The Zn group reached 100% positive animals at WPS 7; the other conduit groups reached this at WPS 8, and one animal in the Aut group remained without a foot extension until later weeks. Overall, the Zn group showed a more consistent early improvement in foot and toe functions than the other groups.

### 3.4. Micro-CT with Iodine Contrast

**Figure 4:**
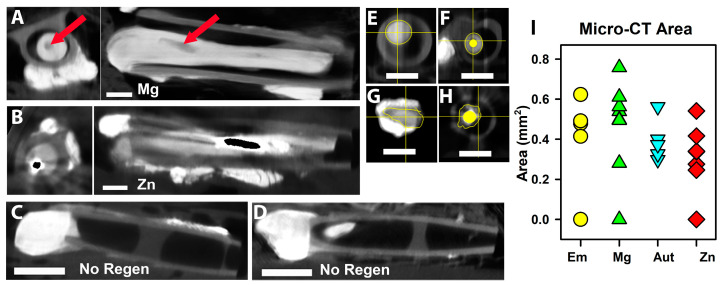
**Micro-CT of nerve tissues with iodine infiltration.** (**A**,**B**) axial (left) and longitudinal (right) images from a Mg (**A**) and a Zn (**B**) animal. Proximal is to the left in all longitudinal sections. The red arrows point to the cavity left by Mg. Centrally located black material in B indicates off-scale pixel values. (**C**,**D**) Images of two animals that did not have tissue regeneration (No Regen). (**E**–**H**) Examples of how tissue area was calculated for the axial slice with the thinnest total area of tissue (not including metal areas). (**I**) Graph of tissue areas, symbols/colors as in Figure 3A.

To examine metal degradation and regenerated tissues, dissected tissues were imaged via micro-CT after immersion in iodine to provide soft tissue contrast. Figure 4A,B are examples of axial sections and longitudinal reconstructions of a Mg and Zn animal, respectively. With Mg, the wires appear gray or black as the metal was replaced with degradation products and then water during tissue processing. Only a few pieces remained (red arrows in Figure 4A), indicating significant degradation. The Zn metal did not disappear and appears to have a black core (off-scale values when the contrast was adjusted to image soft tissues) with dense white material around it, probably degeneration products, and then less-dense tissue outside. Figure 4C,D show images of the conduits of the Em and Mg NoRegen animals, showing the proximal stumps inside the conduits, but no tissue distal to that. To compare between groups, axial sections were chosen at what was estimated to be the thinnest circumference of total tissue, and areas of just tissue were measured. Representative measurements are shown in Figure 4E–H (Em, Mg, Aut, and Zn groups). Results are graphed in Figure 4I. Omitting the NoRegen animals (values of 0), there was only a trend towards significance (ANOVA, *p* = 0.053, n = 4, 7, 5, and 6 for Em, Mg, Aut, and Zn).

### 3.5. Histology

**Figure 5:**
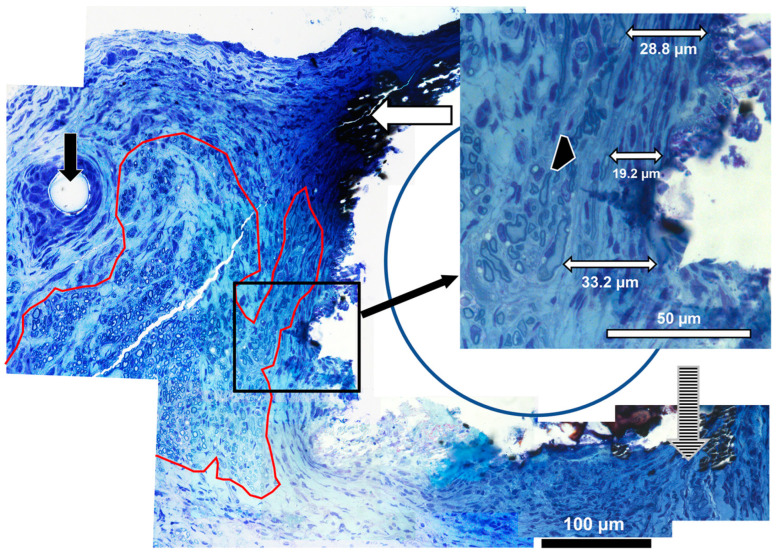
**High-magnification histology.** Axons adjacent to Zn-2%Fe in distal tissues (100× objective). The area outlined in red has clusters of myelinated axons, the vertical hatched arrow shows scattered myelinated axons, and the vertical black arrow points to a suture thread. The horizontal white arrow points to metal degradation products. The insert shows samples of measurements of the capsule, with one measurement to an axon within the capsule (19.2 µm), while the outer edge of the capsule is indicated by the black arrowhead.

Axons in Distal Tissues: Myelinated axons were visualized in distal tissues with toluidine blue staining after preserving myelin. Figure 5 is a high-magnification image of tissues at the edge of a Zn filament, which now appears as a cavity (the metal indicated by a blue circle). An area containing abundant myelinated axons is circled in red, and scattered axons are shown by the cross-hatched arrow. Zn degradation products were densely stained (white horizontal arrow) and often merged into the tissue. The insert shows a higher magnification of the region next to the metal in a place without inflammatory cell deposits. The connective tissue capsule can be identified as circumferential bands of fibroblasts and ECM, presumably mostly collagen. Examples of capsule measurements are shown. The double-headed arrow labeled 19.2 µm is from the inner edge to a myelinated axon in the capsule, and the black arrowhead is the outer edge of the capsule. This image was cut at an oblique angle, so it contains more regions of connective tissues than true cross sections. Reactions to metal can be compared to reactions to an epidural suture (black arrow), which consisted of a thick layer of macrophages and a thick capsule.

**Figure 6:**
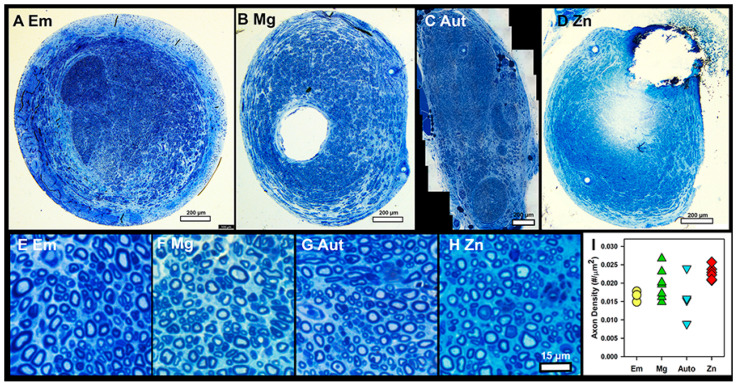
**Axon density in toluidine-blue-stained sections.** (**A**–**D**) Representative low-magnification images from distal nerves of each group. Bars = 200 µm. (**E**–**H**) Representative high-magnification images used for axon density counts. Bar = 15 µm. (**I**) Average axon densities per group, symbols/colors as in Figure 3A.

Axon Density: Axon density was measured in toluidine-blue-stained sections. Figure 6A–D are low-magnification images, and Figure 6E–H are representative high-magnification images used for calculating axon density. Figure 6I is a graph of the average densities per group. There was a significant difference (ANOVA *p* = 0.045, n = 4, 7, 4, 6 for Em, Mg, Aut, Zn), but no differences were detected through a Student–Newman–Keuls post hoc test with *p* < 0.05. However, the Zn group average was higher than the Aut group average at *p* = 0.057 and the Em group average at *p* = 0.063, suggesting a positive trend. No obvious differences in axon diameter, shape, or myelin thickness were detected by five blinded observers (compare Figure 6E–H), so these variables were not quantified.

**Figure 7:**
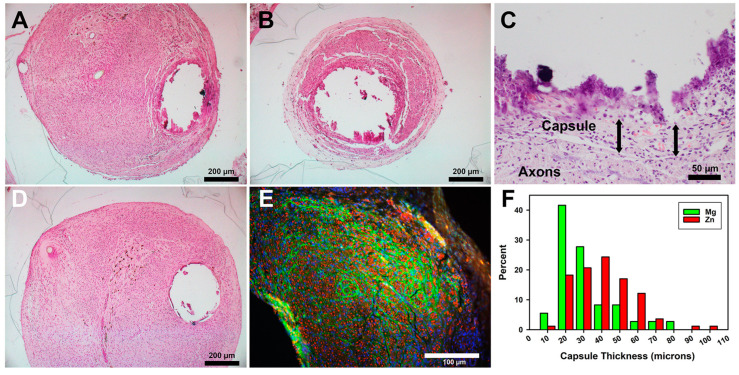
**Histology of paraffin-embedded nerve tissue cross sections.** (**A**–**D**) H&E stain. (**E**) Immunostaining for axons (red) and the perineurium (green). (**A**) A Zn section at the proximal end of conduit. (**B**) A more distal Zn section. (**C**) Higher magnification of the edge of a Zn cavity. Arrows show capsule thickness, both ~50 µm. (**D**) A Mg section at a proximal position. (**E**) Immunostained section of a Zn animal. Bars in A, B, and D = 200 µm; in C = 50 µm; in E = 100 µm. (**F**) Histogram of capsule thickness per metal.

Histology of paraffin-embedded proximal tissues: Packed axons similar to those shown in Figure 6 were also observed in proximal tissues stained with H&E (Figure 7A–D). In Zn animals, there were essentially four layers in the walls of the cavities (Figure 7C). The densest Zn material was lost during tissue processing, leaving a cavity. The materials most central in the cavities were very densely stained, suggesting that they were either degradation products or tissues impregnated with degradation products. The torn appearance of this material suggested that it had been tightly attached to or integrated with the metal. Outside the degradation byproducts but also embedded in them were inflammatory cells that consisted of leukocytes and macrophages, typical of chronic inflammation. Foreign-body giant cells were not observed, although some embedded cells could not be identified due to the dense staining. Some pockets of greater inflammation were seen. Surrounding both degradation materials and inflammation was the connective tissue capsule, described above. The fourth layer, immediately external to the capsule and filling the rest of the nerve, consisted of densely packed axons in fascicles. With the Mg animals, only rare remnants of degradation products or inflammatory cells were found inside the connective tissue capsule (Figure 7D). Tissues were also immunostained for axons (anti-neurofilament protein, red) and a functionally active perineurium (anti-glucose transporter protein-1, green) (Figure 7E). The perineurial fibroblast staining (Figure 7E, green) showed that axons were primarily organized into mini fascicles, a known characteristic of regenerating nerve tissue [37]. Some areas showed the merging of fascicles into larger fascicles (see lower left part of Figure 7E), suggesting nerve maturation, but no consistent differences in fasciculation patterns were detected between groups. The average thickness of the capsules for Mg was 23.3 +/− 8.9 µm (n = 3), and for the Zn alloy it was 35.7 +/− 12.1 µm (n = 5). These were not statistically different (Student’s t-test *p* = 0.176). A histogram of the raw values is shown in Figure 7F. The median, like the average, for the Zn group was slightly higher. Note that all capsule measurements were less than 100 µm. The diameters of the cavities were measured between the inner edges of the capsule tissues to provide a better idea of the metal degradation/resorption. The average diameter was 312.6 +/− 6.6 µm for Mg and 470.3 +/− 73.6 µm for Zn, and these were statistically different (Welch’s t-test *p* = 0.0083). As the original wires were ~300 µm, this suggests that the Mg in these positions had not significantly degraded, while, with Zn, the combined degradation and inflammatory materials appeared to have pushed the nerve tissues away from the central metal by approximately 85 µm (470–300 µm = 170/2 = 85 µm thickness)..

## 4. Discussion

### 4.1. Zn Alloy and Surgical Placement

The Zn alloy used in this study was a proprietary alloy that had shown a similar lower corrosion rate than pure Zn in vitro and after the in vivo implantation of metal pellets (7 mm diameter, 2 mm height) under the skin of rats [30]. This alloy and other Mg-Fe alloys made by our team showed no in vivo toxicity after in vivo implantation [30,31,32]. The current study advances this information by demonstrating biocompatibility and no toxicity for delicate regenerating axons, using filaments of a much smaller mass. The use of electropolishing to produce the final filaments resembles the natural process of degradation, which results in a rough surface with the type of micro- and nano-topology that has been shown to encourage the cellular extension and migration of neurons [38]. While increased surface roughness might have aided neural cell attachment to the metal, this was not seen in a study that compared the implantation of electropolished (rough surface) to anodized (smooth surface) Zn wires and found that the smoother surface resulted in better tissue integration into arterial walls in rats [39]. The complicating factor in all such studies with absorbable metals is that any surface treatment changes both the roughness and the degradation rate, both of which affect tissues. Further work is needed to separate the effects of surface roughness from those that occur due to metal degradation.

To aid surgical placement, the metal filaments were inserted through the walls of the silicone nonpermeable nerve conduits prior to use. This differed from our earlier studies where we inserted the metal filaments into the nerve stumps by hand during surgery and covered the area with permeable polymer conduits attached to the nerve stumps. While silicone conduits are not permeable, which is preferred for nerve conduits, the stiffness of the material was considered beneficial to prevent deformation (and potential breakage) of the metal in vivo. While our earlier studies did not show any displacement of the filaments, and the tissue growth onto and around the filaments was robust, this new, pre-formed scaffold method made surgical application simpler; the metal did move during or after surgery and it provided a more exact distance of the metal–tissue contact. The disadvantage was that the metal was at an angle, which resulted in some challenges in the analysis. However, this did not alter either the tissue attachment to the metal nor our conclusions.

### 4.2. Early Health

Some early health benefits of Zn were suggested, although not definitive, in comparison to the other groups. Most striking was the fact that there were early animal deaths; one in the Aut group and three in the Mg group, and none in the Zn or Em groups. Deaths were attributed to sepsis and multiorgan failure. As we had not encountered such deaths previously, using the same surgical team and procedures, a suspected factor was our use of a combination of an NSAID and an opioid for analgesia, which was novel for our team. Both analgesics can cause adverse effects individually, but the combination of the two has resulted in random rodent deaths [40,41,42,43,44,45]. However, another factor causing animal stress could have been the production of hydrogen bubbles as Mg degraded, as shown in Figure 2F. All stressors combined might have made the animals more susceptible to infection. Interestingly, in terms of infection, Zn ions are known to have significant antibacterial and antisepsis properties [20]. While Mg metal provides some antibacterial effects, its effectiveness in vivo has been questioned [46]. Another indication that Zn may have been beneficial was that fewer Zn animals showed a decrease in weight after surgery. Weight loss in rodents suggests ill health or pain [41]. Finally, the lack of regeneration in one animal each in the Em, Zn, and Mg groups suggests further impediments to regeneration. The cause for the Zn event, but not for the other two, appears to have been that the proximal nerve stump did not remain inside the conduit after surgery, rather than a failure to create a tissue strand. While our data are limited by low animal numbers, it will be interesting to pursue determining whether Zn metal has beneficial effects on animal health.

### 4.3. Functional Recovery

As our experiment involved a noncritical 6 mm gap, it is not surprising that function returned in almost all animals, even in the Em group, our negative control. A critical gap size for rats is around 14–15 mm and 20–25 mm in humans, presumably because fibrin bridges that support cells cannot form in longer gaps [4,9,47,48]. We chose a shorter gap to determine the biocompatibility and safety of the alloy. With a short gap, the timing of functional recovery becomes important for comparing repair strategies. Our data again support but do not prove some positive effects on early function in the Zn group (foot and toe measurements, but not calf circumference). With the pinch test, the least subjective measure, animals in all the conduit groups began responding one week before those in the Aut group. This could be explained if there was a longer lag time required in autografts to remove the cellular and matrix materials before regrowth progressed, compared to only fluid found inside the conduits. Then, it was surprising that toe pinch responses decreased between WPS 15 and 17. This did not appear to be due to ill health, as weights did not decrease. Decreased pinch responses also declined for the skin on the lateral foot, where responses had increased at the same time as the toe responses (Figure S2E). We were aware that sensation on the lateral foot was most likely due to the expansion of the saphenous nerve innervation territory. This nerve normally innervates the medial foot and the toes but expands after injury, with the least expansion to the lateral toe [49]. Our data could be explained by having an expansion of the saphenous nerve territory to both the lateral foot and lateral toe, and then a later decline possibly due to the retraction of saphenous nerve fibers when replaced by regenerating sciatic nerve fibers. Another means of identifying true sensory regeneration might be retrograde tracing.

For toe spread, one of the most subjective scores, the recovery was minor; no animal showed a return to normal toe movement, and a somewhat bimodal pattern of functional return was observed with early signs at WPS 5–10 (the highest in the Aut group): a reduction in toe spread followed by more consistent improvement after WPS 11. However, there were no group differences in the final scores. Toe contractures and foot extensions both appeared abruptly at different time points, and both were assumed to be due to uneven nerve regeneration in one set of opposing muscles but not the other. Leg extensions appeared at WPS 11 (similar to pinch responses), and the Mg and Zn groups had more animals with earlier and higher numbers than the Em and Aut groups. Toe contractures appeared at WPS 5 (similar to toe spread), with a higher percentage of animals in the Zn, Mg, and Em groups than in the Aut group. Note that both contractures and extensions would have significantly interfered in the standard Sciatic Function Index (SFI) and the Static Sciatic Index (SSI) [50,51], which are often used in this field, but which require foot contact with the ground and toe spread. Overall, it is again suggestive but not definitive that Zn had an effect on functional recovery.

### 4.4. Micro-CT Imaging

Micro-CT imaging with iodine contrast, explored previously by our lab [14,34,35], allowed the rapid identification of animals that had not developed intact tissue strands across the gap, and showed that the Mg, but not Zn, filaments had degraded to the point of having gaps. Imaging also showed that regenerating tissues in both the Zn and Mg groups attached to and closely surrounded the metal and crossed the conduit gaps by traveling along the metal. This demonstrates excellent biocompatibility and support of the cells. Analysis of the tissue areas around the filaments, although difficult due to resolution issues and artifacts with the Zn filaments, showed no significant differences, as with the functional outcomes.

### 4.5. Axon Density

Axon density is one measure of the successful regeneration and quality of tissues. Here, we show no significant differences in axon density between the groups, thus showing that while Zn presence did not stimulate an increase in density, it did not result in a loss of density. Axon density is a measure of both axon fiber size and the amount of connective tissues around and between the axonal fibers. Visual inspection confirmed that neither Mg nor Zn stimulated a significant accumulation of inflammatory or fibrotic tissues between axons.

### 4.6. Histology

The histology of the tissues around the metal filaments revealed that the Zn alloy generated more degradation byproducts and chronic inflammation than pure Mg. Despite this difference, both Mg and Zn wires were encapsulated with a similar thickness of connective tissues, suggesting that, at this 4.25-month timepoint, the Zn materials had not proven to be aggravating enough to stimulate a significantly greater encapsulation than Mg. The inflammation with Zn was typical of a chronic inflammation with leukocytes and numerous macrophages. The presence of abundant inflammatory cells in the degradation products and the lack of foreign-body giant cells suggest a strong and ongoing cellular participation in degradation, without the need for macrophage fusion into giant cells to accomplish material degradation. The reaction to Mg was quite minimal, as we had seen previously [12,13]. With both metals, there was no extensive tissue toxicity or excessive inflammation or other adverse tissue responses.

Our histological observations are like those reported by others, especially those of the Goldman group, who pioneered an innovative animal model to study absorbable metals for potential use in vascular stents by implanting metal wires of 250 µm diameters in the walls of rodent abdominal arteries [26,27,52]. For Mg and Mg alloys, their group, as many other groups have, reported excellent biocompatibility with low inflammation and very little retention of byproducts [11,53,54,55,56,57]. However, the low strength and rapid degradation made Mg a poor candidate for a stent, and we speculated that this was also true for nerve repair [13,14], which is why the Zn-Fe alloy was tested. For pure Fe, reports have shown the excessive production of byproducts that resulted in pushing intact tissues away by ~750 um and stimulated greater inflammatory reactions in vivo than pure Mg [27,52,53,58]. We did not see this extent or type of excessive accumulation of byproducts or inflammation. One concern of this study was that regenerating axons might be particularly sensitive to the potential toxicity of the Fe content, even though our team had previously shown no toxicity, inflammation, or changes in blood parameters, and no abnormalities in other organs after the implantation of much larger pellets of Zn-Fe alloys under the skin of rodents [30,31,32]. We can now add that this Zn-2%Fe alloy is not detrimental to nerve regeneration.

With Zn and Zn alloys, others have shown similar histological responses to wires implanted in rat arteries in vivo. This includes, centrally, the metal, then dense degradation materials, then inflammatory cells, then a capsule, and then relatively normal vessel tissues [26,27,59,60,61,62,63,64]. The Goldman group studied several Zn alloys and, in general, the biological responses were less favorable than those with pure Zn [27]. However, they also observed that, with Zn alloys that corroded more rapidly than very pure Zn, the degradation products appeared looser, allowing earlier and greater infiltration by macrophages, which then resulted in better functional outcomes [33]. As we did not test pure Zn, we were not able to explore these aspects. However, we did see the significant penetration of cells into the degradation products with this Zn alloy.

Reactions to metal implants in nervous tissues have been extensively studied because of the use of metal electrodes to record electrical activity. With traditional electrodes placed in CNS or PNS tissues, activated glial cells form scars around the electrodes that, by around six to eight weeks after implantation, push neurons and myelinated fibers away from the metals by between one hundred and several hundred µm, resulting in a degradation of recordings [65,66,67]. In our study, after seventeen weeks, the connective tissue capsules were all below 100 µm for both types of metals. However, with Zn, the degradation products and inflammatory tissues did push tissues away from the solid core. In other studies, Zn wires of 250 µm retained ~60% of their material after 24 months in artery tissues [26,59], so we can assume that the Zn core material remained around 300 µm. As the diameter of the cavities averaged 470 µm, the thickness of the degradation plus the inflammatory materials was ~85 µm (as outlined previously). Adding this to the ~35 um of the capsule, this meant that the nerve fibers were around 120 um away from the densest Zn. That is comparable to the retreat of nerve tissue around an electrode, although the nature of the materials surrounding the Zn alloy was very different from those around the electrodes (not a glial scar). Thus, we suggest that the Zn alloy did not cause the excessive retraction of the nerve tissues, again showing that this Zn-2%Fe alloy is nontoxic to delicate, regenerating nerve tissues and is promising as a biomedical implant material for delicate, regenerating nervous tissues.

## 5. Conclusions

Our data support the following conclusions:

1. Zn-2%Fe and pure Mg filaments placed inside silicone nerve conduits supported cellular attachment to both metals and the regeneration of sciatic nerves across a 6 mm, noncritical injury gap in adult rats.
2. Zn-2%Fe filaments were well tolerated and had fewer adverse effects than Mg filaments, empty conduits, or autograft controls during early recovery from surgery.
3. All groups supported the regeneration of equal densities of axons distal to the injury site and equal recovery of functions, although the Zn group showed a trend towards an increase in earlier recovery of function.
4. The Zn-2%Fe filaments showed a greater local accumulation of degradation products and inflammatory cells than Mg filaments, but no difference in connective tissue capsule thickness and no toxicity or significant inflammation in newly regenerated nerve tissues.

The exciting aspects of this work are the excellent biocompatibility with nerve tissues of both the Zn alloy and Mg. While there were no definitive signs of functional improvements, the future of nerve repair strategies is moving towards combinations of therapies rather than single therapies. The benign absorbable metals could provide the physical guidance that can then be readily combined with other strategies to stimulate nerve outgrowth to achieve excellent functional return. Also intriguing and worthy of further exploration is to investigate how released Zn metal ions might aid early animal health. The limitations of this study were the small animal numbers and the short gap distance tested, which limited the analysis of the functional return, but was crucial to show biocompatibility. Future directions could include using the strength of an absorbable metal scaffolding to physically support nerves and tissues after traumatic injuries that cause extensive tissue damage and require the reconstruction of large tissue blocks. Another potential use of thin metal filaments could be to direct axons into one of several downstream directions at axon branching. Another might be to create pathways for axon growth through dense white matter tracts in the CNS or through glial scars. Further, in the CNS, regeneration issues appear if the materials used for physical guidance across injuries are so attractive to axons that the axons do not detach appropriately and therefore do not integrate into the target tissues; axons cannot remain attached if the metal disappears. Overall, we believe that there are multiple ways in which absorbable, nerve-compatible metals could improve nerve tissue regeneration after injury.

## Data Availability

No new data were created or analyzed in this study. Data sharing is not applicable to this article.

## Supplementary Materials

The following supporting information can be downloaded at: https://www.mdpi.com/article/10.3390/pharmaceutics15112595/s1, Figure S1: Functional measurements; Table S1: Functional analysis; Figure S2: Raw function scores; Table S2: Animal numbers; Figure S3: Animal weights.
