# Supplementary material for: Nerve Regeneration with a Scaffold Incorporating an Absorbable Zinc-2% Iron Alloy Filament to Improve Axonal Guidance
Source: Pharmaceutics. 2023 Nov 7;15(11):2595. doi: 10.3390/pharmaceutics15112595 (PMC10674795; doi:10.3390/pharmaceutics15112595)
Supplement: Supplementary file 1 [file pharmaceutics-15-02595-s001.zip › pharmaceutics-2666790-supplementary.pdf]

Supplementary Information

Nerve Regeneration with a Scaffold Incorporating an Absorbable Zinc-2% Iron Alloy Filament to Improve Axonal Guidance

Tomer Ron, Avi Leon, Alon Kafri, Ahmed Ashraf, John Na, Ashvin Babu, Runima Banerjee, Hunter Brookbank, Saimahesh Raju Muddaluri, Kevin J. Little, Eli Aghion and Sarah Pixley

Figure S1. Functional Measurements

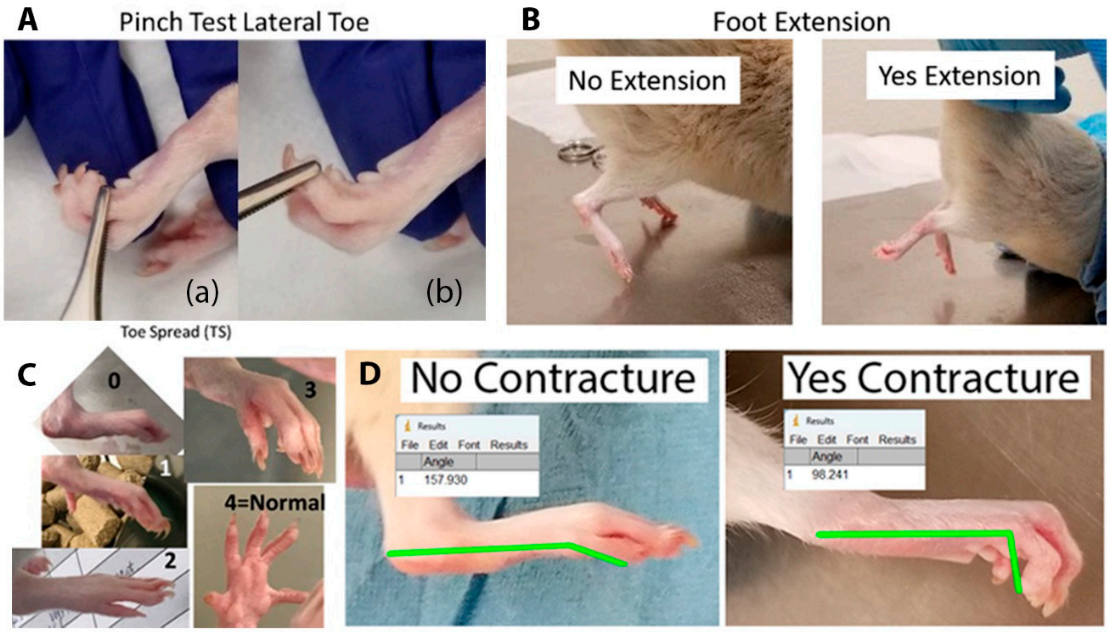

Figure S1. Functional Measurements. A) Analysis of sensory function was done by a pinch test, with a focus on the lateral hind toe. Pinch area 1 was on the phalanx, area 2 was the tip of the toe. B) Foot Extension was determined as positive if the foot was fixed at an angle of >90 degrees relative to the calf (tibia and fibula bones). If movement was free and held primarily at or less than 90 degrees, this was considered no extension. C) Toe Spread was a score of how much voluntary movement there was of the lateral toe relative to the rest of the foot. No animal had greater extension than a 3 and none reached normal toe movement that includes both extension laterally and dorsally (arbitrarily labeled a score of 4, but no animal had that score and it was not used). D) In ImageJ using the angle function, a first line was made from the back of the ankle to the bony knuckle at the base of the lateral toe. The second was from there to the innermost part of the pad on the lateral toe.

Table S1: Functional Analysis

Functional Scales	
**Pinch of Lateral Toe #5**	**Toe Spread (TS)**
0 = No response to pinch on lateral toe	0 = No movemnt
1 = Response to one of two spots on toe	1 = Slight movement of lateral toe laterally
2 = Response at both spots on lateral (Normal)	2 = Definite lateral movement and gap opening
**For % in graphs: Positive Pinch = > 0**	3 = Greater lateral movement with some upward
	10 = Normal (no animal reached normal)
	**For % in graphs: Positive TS = Scores 2-3**
**Foot Extension Scale**	**Toe Contracture Angle**
1 = 90 (normal) and < 90 degrees	Measured angle of two lines:
2 = > 90 degrees	From ankle of foot to knuckle at base of lateral toe
3 = More Extension > 90 than 2	From knuckle to innermost point of lateral toe pad
4 = More Extension >90 deg than 3	Normal was in line with foot, >120 degrees.
5 = Most Extension, almost 180 deg	**For % in graphs: Positive Contracture = < 120 degrees**
**For % in graphs: Positive Extension = Scores 2-5**	
**Pinch of Lateral Foot**	
0 = No response to pinch of skin	
1 = Response to pinch of skin at back of ankle	
2 = Previous plus skin on lateral, posterior side of foot	
3 = Previous plus skin on lateral, anterior side of foot	

Table S1. Functional Analysis. This table gives the description of the functional scores in words.

Figure S2. Raw Function Scores

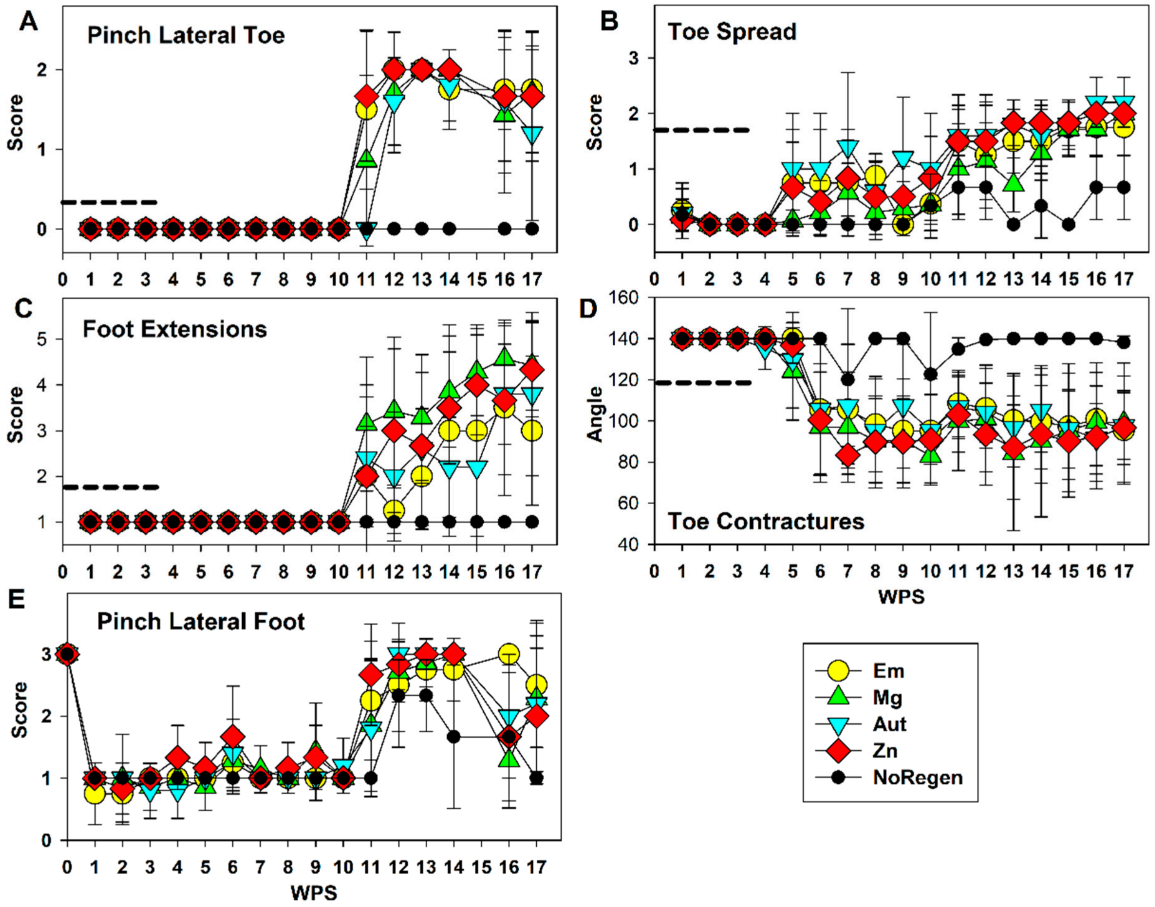

Figure S2. Raw Function Scores. The raw scores averaged per group are shown for each function, including the Pinch test at the ankle and along the lateral foot (E). Almost all animals reacted to a pinch of the skin at the back of the ankle after injury. The black dashed lines in A – D indicate the cut-off values. Values per WPS that were above the lines for A, B, C and below the line for Toe Contractures (D) were considered positive (Yes). To obtain the Graph in Figure 3, a positive or negative value was calculated per animal per timepoint and then the percent of positive animals per group per timepoint was calculated and graphed.

Table S2. Animal Numbers

Columns:	1	2	3	4	5	6	7
**Numbers**	**Surgery n**	**# Dead**	**# <95 W%**	**# No Regen**	**# No Adverse**	**n Surviving**	**n For Analyses**
**Em**	5	0	2	1	2	5	4
**Mg**	11	3	4	1	3	8	7
**Aut**	6	1	1	0	4	5	5
**Zn**	7	0	0	1	6	7	6
**Percents**	**Surgery n**	**% Dead**	**% <95 W%**	**% No Regen**	**%No Adverse**		
**Em**	5	0.0	40.0	20.0	40.0		
**Mg**	11	27.3	36.4	9.1	27.3		
**Aut**	6	16.7	16.7	0.0	66.7		
**Zn**	7	0.0	0.0	14.3	85.7		

Table S2: Animal Numbers. Animal Numbers are given in the top set of rows and percent per group in the bottom set.
Columns:

1. Surgery n is the number of rats after surgery.
2. The # or % Dead is the number/percent per group that died during the first week after surgery.
3. The # or % <95% is the number of rats that lost more than 5% of their body weight in the first week after surgery. This variable did not affect final numbers of animals for analyses.
4. The # or % No Regen is the number that lacked an intact tissue strand in the conduit after 17 WPS.
5. The # or % No Adverse is the number/% of rats per group that did not have any of the events in all in Columns 2-4.
6. The n Surviving is the number of rats surviving after the first week, which were used for analysis of function.
7. The n For Analyses is the previous number minus the rats without regeneration, which was the number available for certain analyses.

Figure S3: Animal Weights

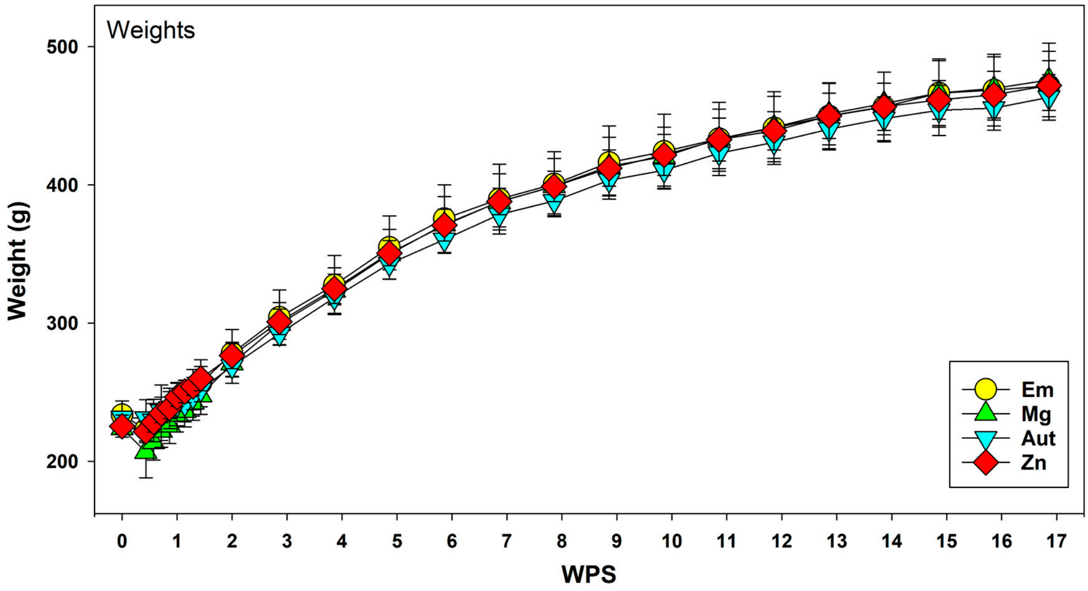

Figure S3. Animal Weights. Averages per group of animal weights per week post-surgery (WPS). No statistically significant differences were seen between groups at any time point.
